# Examining Transparency in Kidney Transplant Recipient Selection Criteria: Nationwide Cross-Sectional Study

**DOI:** 10.2196/74066

**Published:** 2025-11-04

**Authors:** Belen Rivera, Stalin Canizares, Gabriel Cojuc-Konigsberg, Olena Holub, Alex Nakonechnyi, Ritah R Chumdermpadetsuk, Keren Ladin, Devin E Eckhoff, Rebecca Allen, Aditya Pawar

**Affiliations:** 1 Beth Israel Deaconess Medical Center Boston, MA United States; 2 Department of Computer Science and Mathematics Mount St Joseph University Cincinnati, OH United States; 3 Department of Community Health Research on Aging, Ethics, and Community Health (REACH Lab) Tufts University Boston, MA United States

**Keywords:** kidney transplant, kidney transplant recipient, selection criteria, artificial intelligence, AI, large language model, natural language processing

## Abstract

**Background:**

Choosing a transplant program impacts a patient’s likelihood of receiving a kidney transplant. Most patients are unaware of the factors influencing their candidacy. As patients increasingly rely on online resources for health care decisions, this study quantifies the available online patient-level information on kidney transplant recipient (KTR) selection criteria across US kidney transplant centers.

**Objective:**

We aimed to use natural language processing and a large language model to quantify the available online patient-level information regarding the guideline-recommended KTR selection criteria reported by US transplant centers.

**Methods:**

A cross-sectional study using natural language processing and a large language model was conducted to review the websites of US kidney transplant centers from June to August 2024. Links were explored up to 3 levels deep, and information on 31 guideline-recommended KTR selection criteria was collected from each transplant center.

**Results:**

A total of 255 US kidney transplant centers were analyzed, comprising 10,508 web pages and 9,113,753 words. Among the kidney transplant guideline–recommended KTR selection criteria, only 2.6% (206/7905) of the information was present on the transplant center web pages. Socioeconomic and behavioral criteria were mentioned more than those related to the patient’s medical conditions and comorbidities. Of the 31 criteria, finances and health insurance was the most frequently mentioned, appearing in 25.5% (65/255) of the transplant centers. Other socioeconomic and behavioral criteria, such as family and social support systems, adherence, and psychosocial assessment, were addressed in less than 4% (9/255) of the transplant centers. No information was found on any web page for 45.2% (14/31) of the KTR selection criteria. Geographically, disparities in reporting were observed, with the South Atlantic division showing the highest number of distinct criteria, while New England had the fewest.

**Conclusions:**

Most transplant center websites do not disclose patient-level KTR selection criteria online. The lack of transparency in the evaluation and listing process for kidney transplantation may limit patients in choosing their most suitable transplant center and successfully receiving a kidney transplant.

## Introduction

### Background

Kidney transplantation is considered the gold standard treatment for patients with end-stage kidney disease [1-3]. However, to receive a kidney transplant, a patient must first be referred, evaluated, deemed a suitable candidate, and then listed at a transplant center [4,5]. Patients may not be fully aware of the factors that shape their candidacy for a kidney transplant or of the variation across centers in reliance on certain evaluation criteria [1,6]. Selecting a transplant program is a critical decision, with far-reaching implications that impact the success of receiving a transplant [5].

Reports from the Organ Procurement and Transplantation Network Ethics Committee on transparency in program selection; the National Academies of Sciences, Engineering, and Medicine on advancing equity in transplantation; and the Health Resources and Services Administration initiatives emphasize the importance of accessible information in supporting shared decision-making [7,8]. In addition to the recognized lack of transparency in available information, there is notable heterogeneity in the prewaitlisting practices and kidney transplant recipient (KTR) selection criteria reported by transplant centers [5,7,9-11]. These inconsistencies contribute to inefficiencies and inequities impacting waitlisting and eventual transplantation, especially considering that insurance plans may only cover a single transplant evaluation [7].

Despite the evolving landscape of policies aimed at increasing equity and access in the prewaitlist process, such as the Increasing Organ Transplant Access (IOTA) model, a critical information gap remains in quantifying the extent of the unavailable information regarding KTR selection criteria, which helps guide the construction of these policies [12]. Furthermore, in an era where patients increasingly use online resources for health care decision-making, accessible online patient-level KTR selection criteria information may aid patients in selecting their most suitable transplant program [13-16]. Recurrent themes have been expressed by patients regarding the difficulty in finding patient-centered information and the lack of transparency throughout the kidney transplant process, which hinder their ability to make informed decisions [17-19].

### This Study

While the criteria for determining priority in the national organ allocation system are explicit, those for determining transplant candidacy at the transplant center level remain unclear for most patients. Fragmented data, an overwhelming volume of information across nationwide programs, and their various website links can be challenging for human evaluators to navigate. Moreover, the dynamic nature of online content further complicates the evaluation process. Therefore, using artificial intelligence (AI), specifically training a large language model (LLM), has emerged as a valuable tool to automate this analysis, making it comprehensive, scalable, and efficient.

In this study, we aimed to use natural language processing (NLP) and an LLM to quantify the available online patient-level information regarding guideline-recommended KTR selection criteria reported by US kidney transplant centers.

## Methods

**Figure 1:**
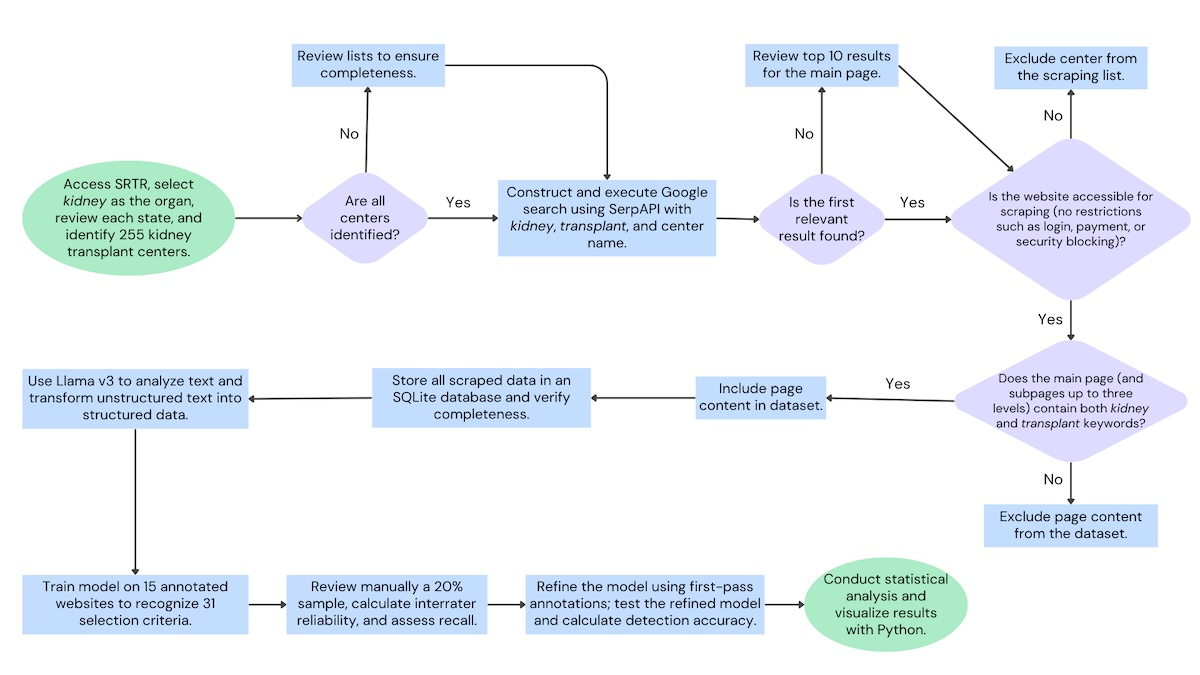
Overview of data aggregation, scraping, cleaning, and analysis. SRTR: Scientific Registry of Transplant Recipients.

As a brief overview of the study’s methods, we determined guideline-recommended KTR selection criteria [20], aggregated data from US kidney transplant center websites, and trained and refined an LLM to analyze the available information. The method sequence is illustrated in Figure 1.

### Ethical Considerations

A determination form confirming that the study did not involve human participants was approved by the Beth Israel Deaconess Medical Center Institutional Review Board.

### Selection Criteria Definition

KTR selection criteria categories used in our analysis for the LLM were collected by consulting the Kidney Disease: Improving Global Outcomes Clinical Practice Guideline on the Evaluation and Management of Candidates for Kidney Transplantation [20]. This guideline is designed to aid decision-making and provides a framework for the KTR selection criteria, all of which were included in our list. In addition, to broaden the scope of possible selection criteria and assess the alignment between LLM and human review in identifying KTR selection criteria, we conducted a manual search of the 15 transplant center websites in the New England division. Frequently mentioned criteria not present in the Kidney Disease: Improving Global Outcomes guidelines were added (Table S1 in Multimedia Appendix 1).

### Data Aggregation

We accessed the Scientific Registry of Transplant Recipients to compile a list of 255 US kidney transplant centers. Specifically, in the search bar, “kidney” was selected as the organ, and then each state was reviewed to include the associated transplant center or centers in our dataset.

To access the transplant centers’ websites, we conducted an online search using Google [21] searches via SerpAPI (SerpApi, LLC) [22] with the keywords “kidney” and “transplant” along with the name of each kidney transplant center and selected the first organic result. We opted to use Google because it is the most common search engine, with an 82% market share worldwide [23]. To ensure a systematic approach, we used SerpAPI, a search engine tool designed to access results programmatically in a structured format [24]. When we refer to a *website*, it indicates the entire online domain associated with a specific kidney transplant center. In contrast, a *web page* refers to a single page within a website. Therefore, for each transplant center, there was one website and multiple web pages.

Each transplant center’s first organic result was manually reviewed. If the results did not appear to represent the center’s main website, the top 10 search results were examined to locate the correct main website. We included text from subpages that were linked to the main website, up to 3 levels in, and to ensure relevance, only pages containing both “kidney” and “transplant” were included in the dataset.

Centers that lacked a clearly identifiable website or appeared only in aggregator listings were excluded from the scraping process. Web pages that required a log in, asked for payment, or disallowed scraping were also excluded (Table S2 in Multimedia Appendix 1).

After defining the web pages to be included, we conducted data scraping for content aggregation. For this purpose, AngleSharp (AngleSharp.IO) [25] was the HTML parser, DotNetBrowser (TeamDev Ltd) [26] was the programmable browser emulator, UglyToad.PdfPig (UglyToad Software Ltd) [27] was used to parse PDFs, and SQLite [28] was used as the database.

### NLP and LLM Analysis

NLP and LLMs enabled the interpretation and computerized *reading* of unstructured text and the transformation of the text into structured data for analysis. Llama (version 3; Meta Platforms Inc) [29] was used to conduct the LLM analysis. Initially, data from 15 manually annotated web pages were used for training the model to identify the 31 predefined KTR selection criteria in the scraped text from all the web pages included.

### Model Testing

Table 1 provides an overview of the model performance metrics.

**Table 1:** Summary of large language model performance metrics during model validation.

Metric	Value	Description
Interrater reliability, %	97.8	Agreement between 2 human reviewers (BR and SC) on 20% of web pages
Recall (approximate), %	99.7	Less than 0.3% of relevant data were missed in the random validation sample
Sensitivity (first-pass model), %	97.6	Accurately identified KTR^a^ selection criteria
Specificity (first-pass model), %	4.2	Low because of false positives (eg, mislabeling “donor” as financial donor)
False positive reduction after refinement, %	54	Improvement in accuracy after further model training
Detection within 95% CI, n/n (%)	23/31 (74)	Number of criteria with detection rates within 95% CI
Detection within 99% CI, n/n (%)	5/31 (16.1)	Number of criteria with detection rates within 99% CI
Criteria exceeding 99% CI, n/n (%)	3/31 (9.7)	GFR^b^, dialysis, and finances and insurance categories exceeded expected detection

^a^KTR: kidney transplant recipient.

^b^GFR: glomerular filtration rate.

#### First Model Testing

A random sample of 20% of the web pages scraped from these first-pass LLM results was then hand-reviewed by 2 investigators (BR and SC). The interrater agreement was high, with 97.8% raw concordance; the corresponding chance-corrected coefficient (Cohen κ=0.39) indicated moderate reliability after accounting for the expected agreement by chance. To facilitate collaborative review and structured comparison of LLM outputs, our team conducted annotation and verification using a shared cloud-based spreadsheet, allowing tabular alignment of criteria across centers and efficient resolution of discrepancies. Discrepancies were resolved through discussion. The human-reviewed sample was used as the ground truth. This first-pass model demonstrated a strong recall within the random sample, missing less than 0.3% of the relevant data. Although the model resulted in a high sensitivity (97.6%), it had an unacceptably low specificity (4.2%). Therefore, we continued to train the model to improve its performance.

#### Second Model Validation

The primary reason that the first-pass–trained version of the model had a high number of false positives was that it tended to misclassify word mentions as KTR selection criteria. For instance, mentioning diabetes on a website does not necessarily mean that it is a transplant criterion. Therefore, to refine the specificity of the model, the annotated sample from the first-pass training was used to further train the model.

Training the model using the additional hand-annotated data resulted in highly accurate estimates of the presence of the 31 criteria across the transplant centers’ websites. Detection estimates were calculated with both 95% and 99% CIs. For 23 (74.2%) categories, detection rates fell within the 95% CI, while 5 (16.1%) categories met the 99% confidence level. However, 3 (9.7%) categories exceeded the upper bound of the 99% CI: glomerular filtration rate (by 35%), dialysis (by 18%), and finances and health insurance. A further refinement of the finances and health insurance category reduced false positives by 54%, largely because of the model’s misinterpretation of the word “donor” as a financial donor rather than an organ donor.

In addition to assessing the overall availability of the kidney transplant center KTR selection criteria, we further examined the clarity and consistency of the criteria presented online by selecting 6 criteria and extracting 2 representative quotes for each, which were then compiled into Table S3 in Multimedia Appendix 1. This table was used to illustrate the heterogeneity and lack of clarity in how these criteria were described across the websites.

Statistical analysis was conducted using Python version 3.12 Matplotlib, Pandas, and Scikit-learn libraries. GeoPandas was used to map the centers based on the ZIP code, and geographic divisions were based on the US census divisions. Each division included a group of states.

## Results

We identified 255 US kidney transplant centers. Of these, we successfully scraped 203 (79.6%) transplant center websites, exploring subpages up to 3 levels deep. The final dataset comprised 10,508 web pages and 9,113,753 words.

### Criteria Mentions by Center

An analysis of publicly available websites revealed that most transplant centers included only a limited range of KTR selection criteria. Across the 255 US kidney transplant centers, we evaluated the presence of 31 guideline-recommended criteria per center, with 7905 expected mentions of the KTR selection criteria. Of these, only 206 (2.6%) KTR selection criteria were found, indicating that only a small fraction of the expected information was publicly available online. Moreover, among the 203 (79.6%) transplant center websites that were successfully scraped, most kidney transplant centers had minimal or no accessible information regarding their transplant criteria; 56.1% (143/255) of the centers did not provide any listing criteria information. A smaller number of centers provided limited information, with 53 (20.8%), 38 (14.9%), and 13 (5.0%) centers listing one, 2, and 3 criteria, respectively. Few centers included information about 4 or more criteria, with 4 (1.6%) and 4 (1.6%) centers listing 4 or more than 4 criteria, respectively. No center listed more than 6 criteria on their website.

### Criteria Mentions by Category

Of all 31 KTR selection criteria included, 14 (45.2%) were not mentioned on any of the 203 transplant center websites analyzed. These included 2 criteria under the socioeconomic and behavioral category (homelessness or other unstable living conditions and support networks), 10 criteria related to medical conditions and comorbidities (diabetes, heart disease, neurocognitive disorder/dementia, hematologic disorders, multiple organ failure, and immunologic assessment), 1 laboratory value (creatinine clearance), and 1 functional status criterion (mobility), which was classified under the category of others.

Conversely, the most common category mentioned was socioeconomic and behavioral in 27.1% (69/255) of the centers (Table 2). Of note, this analysis focused solely on whether the KTR selection criteria were mentioned on transplant center websites, without evaluating the clarity, context, or depth of explanation. Specifically, finances and health insurance were mentioned at 25.5% (65/255) of centers, while a few centers considered other factors—2% (5/255) addressed family and/or social support system, 1.2% (3/255) listed adherence, and 0.4% (1/255) mentioned psychosocial assessments. Homelessness and support networks were not mentioned in any center. Medical conditions and comorbidities were addressed by 47 (18.4%) centers, mainly chronic kidney disease stage 15 (5.9%), life-threatening diseases 12 (4.7%), HIV 8 (3.1%), infection 5 (2.0%), frailty 5 (2.0%), drug or alcohol abuse 4 (1.6%), malignancy 4 (1.6%), smoking 3 (1.2%), and psychiatric illness 1 (0.4%). None of the centers mentioned diabetes, heart disease, peripheral vascular disease, bone or mineral disorders, liver disease, lung disease, neurocognitive disorders or dementia, hematologic disorders, multiple organ failure, or immunological assessments. For demographics and laboratory values, 45 (17.6%) centers assessed at least one criterion. Glomerular filtration rate was the most common (34/255, 13.3%), followed by age (10/255, 3.9%) and BMI (9/255, 3.5%).

**Table 2:** Kidney transplant recipient (KTR) selection criteria availability on transplant center websites (N=255).

KTR selection criteria			Centers with accessible KTR selection criteria, n (%)		Centers without accessible KTR selection criteria, n (%)
**Socioeconomic and behavioral factors**			69 (27.1)		186 (72.9)
	Finances and health insurance	65 (25.5)		190 (74.5)	
	Family and social support system	5 (2.0)		250 (98.0)	
	Adherence	3 (1.2)		252 (98.8)	
	Psychosocial assessment	1 (0.4)		254 (99.6)	
	Homeless or other unstable living conditions	0 (0.0)		255 (100.0)	
	Support networks	0 (0.0)		255 (100.0)	
**Medical conditions and comorbidities**			47 (18.4)		208 (81.6)
	CKD^a^ stage	15 (5.9)		240 (94.1)	
	Life-threatening diseases	12 (4.7)		243 (95.3)	
	HIV	8 (3.1)		247 (96.9)	
	Infection	5 (2.0)		250 (98.0)	
	Frailty	5 (2.0)		250 (98.0)	
	Drug or alcohol abuse	4 (1.6)		251 (98.4)	
	Malignancy	4 (1.6)		251 (98.4)	
	Smoking	3 (1.2)		252 (98.8)	
	Psychiatric illness	1 (0.4)		254 (99.6)	
	Diabetes	0 (0.0)		255 (100.0)	
	Heart disease	0 (0.0)		255 (100.0)	
	Peripheral vascular disease	0 (0.0)		255 (100.0)	
	Bone and mineral disorders	0 (0.0)		255 (100.0)	
	Liver disease	0 (0.0)		255 (100.0)	
	Lung disease	0 (0.0)		255 (100.0)	
	Neurocognitive disorder/dementia	0 (0.0)		255 (100.0)	
	Hematologic disorders	0 (0.0)		255 (100.0)	
	Multiple organ failure	0 (0.0)		255 (100.0)	
	Immunologic assessment	0 (0.0)		255 (100.0)	
**Demographics and laboratory values**			45 (17.6)		210 (82.4)
	GFR^b^ (mL/min)	34 (13.3)		221 (86.7)	
	Age (years)	10 (3.9)		245 (96.1)	
	BMI (kg/m^2^)	9 (3.5)		246 (96.5)	
	Creatinine clearance (mL/min)	0 (0.0)		255 (100.0)	
**Others**			22 (8.6)		233 (91.4)
	Dialysis	22 (8.6)		233 (91.4)	
	Mobility	0 (0.0)		255 (100.0)	

^a^CKD: chronic kidney disease.

^b^GFR: glomerular filtration rate.

When evaluating the availability of the KTR selection criteria for kidney transplant centers on patient-accessible websites, we compiled representative quotes to illustrate how the KTR selection criteria were presented online (Table S3 in Multimedia Appendix 1). These quotes highlight that, in addition to the fact that the KTR selection criteria were mentioned in only 2.6% (206/7905) of instances, many were unclear and inconsistently described.

### Geographic Variation in Transparency

As shown in Table 3, the availability of distinct categories of information across the geographic divisions varied significantly; information gaps were evident across multiple geographic regions. The South Atlantic division (Delaware, Florida, Georgia, Maryland, North Carolina, South Carolina, Virginia, District of Columbia, West Virginia) and the East North Central division (Illinois, Indiana, Michigan, Ohio, Wisconsin) provided the highest percentage of distinct KTR selection criteria, with both divisions showing 41.9% (13/31) availability. In contrast, the West North Central (Iowa, Kansas, Minnesota, Missouri, Nebraska, North Dakota, South Dakota), East South Central (Alabama, Kentucky, Mississippi, Tennessee), and New England (Connecticut, Maine, Massachusetts, New Hampshire, Rhode Island, Vermont) divisions offered the least amount of information, each with only 16.1% (5/31) of the distinct criteria available. Other divisions, such as the Pacific (Alaska, California, Hawaii, Oregon, Washington) and Middle Atlantic (New Jersey, New York, Pennsylvania), displayed moderate information availability at 35.5% (11/31), while the Mountain division (Arizona, Colorado, Idaho, Montana, Nevada, New Mexico, Utah, Wyoming) followed closely with 32.3% (10/31). Despite having numerous centers, the West South-Central division (Arkansas, Louisiana, Oklahoma, Texas) had relatively low information availability, with only 25.8% (8/31) of the distinct criteria found (Table 3).

**Table 3:** Geographical distribution of online information on kidney transplant recipient selection criteria in the US division and state.

Geographic division		Total centers, n		Total expected criteria, n		Total criteria found^a^, n (%)		Average of criteria per center		Distinct criteria found^b^, n (%)
**East North Central**		36		1116		32 (2.9)		0.89		12 (38.7)
	Illinois	10	310		14 (4.5)		1.4		9 (29.0)	
	Indiana	3	93		0 (0)		0.0		0 (0)	
	Michigan	10	310		5 (1.6)		0.5		4 (12.9)	
	Ohio	9	279		8 (2.9)		0.9		6 (19.4)	
	Wisconsin	4	124		5 (4.0)		1.6		5 (16.1)	
**East South Central**		15		465		9 (1.9)		0.6		5 (16.1)
	Alabama	3	93		0 (0)		0.0		0 (0)	
	Kentucky	3	93		5 (5.4)		1.7		4 (12.9)	
	Mississippi	1	31		2 (6.4)		2.0		2 (6.5)	
	Tennessee	8	248		2 (0.8)		0.2		2 (6.5)	
**Middle Atlantic**		39		1209		37 (3.1)		0.95		11 (35.5)
	New Jersey	5	155		13 (8.4)		2.6		6 (19.6)	
	New York	15	465		11 (2.4)		0.7		7 (22.6)	
	Pennsylvania	19	589		13 (2.2)		0.7		7 (22.6)	
**Mountain**		17		527		17 (3.2)		1.00		10 (32.3)
	Arizona	7	217		4 (1.8)		0.6		3 (9.7)	
	Colorado	4	124		3 (2.4)		0.8		3 (9.7)	
	New Mexico	2	62		3 (4.8)		1.5		2 (6.5)	
	Nevada	1	31		3 (9.7)		3.0		3 (9.7)	
	Utah	3	93		4 (4.3)		1.3		3 (9.7)	
	Idaho	0	0		0 (0)		0.0		0 (0)	
	Montana	0	0		0 (0)		0.0		0 (0)	
	Wyoming	0	0		0 (0)		0.0		0 (0)	
**New England**		15		465		9 (1.9)		0.60		4 (12.9)
	Connecticut	2	62		0 (0)		0.0		0 (0)	
	Massachusetts	9	279		8 (2.9)		0.9		4 (12.9)	
	Maine	1	31		0 (0)		0.0		0 (0)	
	New Hampshire	1	31		1 (3.2)		1.0		1 (3.2)	
	Rhode Island	1	31		0 (0)		0.0		0 (0.0)	
	Vermont	1	31		0 (0)		0.0		0 (0.0)	
**Pacific**		30		930		29 (3.1)		0.97		11 (35.5)
	California	21	651		22 (3.4)		1.1		11 (35.5)	
	Hawaii	1	31		2 (6.4)		2.0		2 (6.5)	
	Oregon	3	93		2 (2.2)		0.7		2 (6.5)	
	Washington	5	155		3 (1.9)		0.6		2 (6.5)	
	Alaska	0	0		0 (0)		0.0		0 (0)	
**South Atlantic**		41		1271		47 (3.7)		1.15		13 (41.9)
	District of Columbia	3	93		5 (5.4)		1.7		3 (9.7)	
	Delaware	2	62		3 (4.8)		1.5		3 (9.7)	
	Florida	13	403		15 (3.7)		1.2		7 (22.6)	
	Georgia	4	124		4 (3.2)		1.0		2 (6.5)	
	Maryland	3	93		0 (0)		0.0		0 (0.0)	
	North Carolina	5	155		13 (8.4)		2.6		8 (25.8)	
	South Carolina	3	93		1 (1.1)		0.3		1 (3.2)	
	Virginia	6	186		5 (2.7)		0.8		3 (9.7)	
	West Virginia	2	62		1 (1.6)		0.5		1 (3.2)	
**West North Central**		24		744		11 (1.5)		0.46		5 (16.1)
	Iowa	4	124		2 (1.6)		0.5		2 (6.5)	
	Kansas	1	31		2 (6.4)		2.0		2 (6.5)	
	Minnesota	4	124		1 (0.8)		0.2		1 (2.2)	
	Missouri	10	310		5 (1.6)		0.5		4 (12.9)	
	North Dakota	2	62		0 (0)		0.0		0 (0)	
	Nebraska	1	31		1 (3.2)		1.0		1 (3.2)	
	South Dakota	2	62		0 (0)		0.0		0 (0)	
**West South Central**		38		1178		15 (1.27)		0.39		7 (22.6)
	Arkansas	2	62		0 (0)		0.0		0 (0)	
	Louisiana	4	124		5 (4.0)		1.2		3 (9.7)	
	Oklahoma	4	124		0 (0)		0.0		0 (0)	
	Texas	28	868		10 (1.2)		0.4		7 (22.6)	

^a^This column illustrates the number of times the selection criteria were mentioned on the transplant center websites in a specific division.

^b^Indicates the number of unique selection criteria referenced on the web pages in that division.

States such as Idaho, Montana, and Wyoming, which are part of the Mountain division, do not have kidney transplant centers; therefore, they were not expected to have available KTR selection criteria. North and South Dakota typically rely on regional transplant programs, which may contribute to the lower availability of online KTR selection criteria observed in the West North Central division.

**Figure 2:**
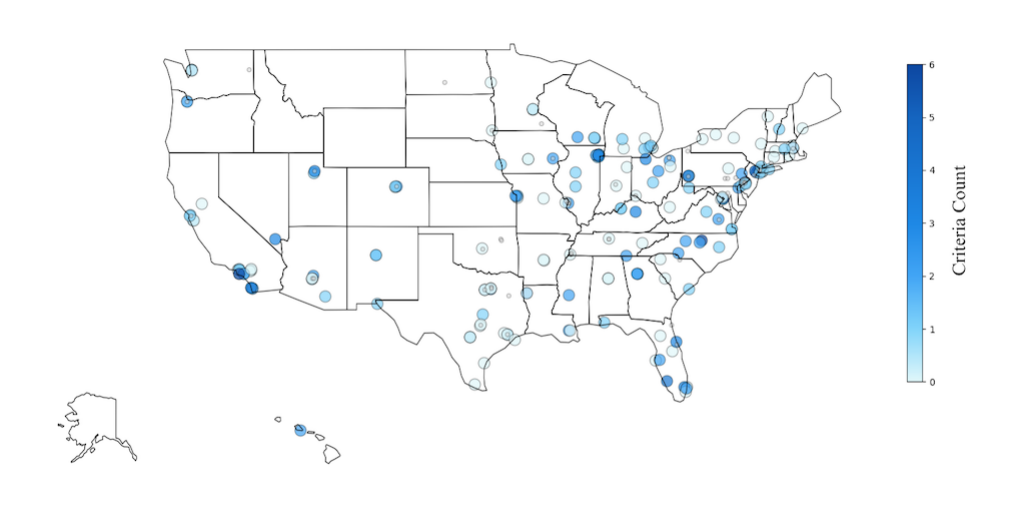
Geographic distribution of US kidney transplant centers by the number of kidney transplant recipient selection criteria mentioned on websites.

The geographic distribution of the individual kidney transplant centers across the US further highlights differences with transparency in reporting KTR selection criteria. A national map of the 255 kidney transplant centers shows each center color-coded by the number of KTR selection criteria mentioned on its website. The scale ranges from 0 to 6, where lighter shades indicate centers with no or few criteria reported, while darker shades represent centers providing more detailed information. Smaller gray markers correspond to centers without available information or those that did not pass initial filtering (Figure 2).

## Discussion

### Principal Findings

In this study, we evaluated patient-facing information regarding KTR selection criteria across the websites of US kidney transplant centers using AI and an LLM. Our findings revealed an unmet need for patients undergoing the kidney transplant prewaitlist process: most transplant center websites with online patient-facing information (7699/7905, 97.4%) did not contain KTR selection criteria, and among those that did, few included information about multiple criteria. More than half of the transplant centers (143/255, 56.1%) did not report any KTR selection criteria, limiting access to crucial patient information. Moreover, variation in the availability of KTR selection criteria information was observed across geographic divisions, with some regions providing more information than others.

These gaps in access to online patient-level information are especially important given the growing reliance on the internet as a tool for health care decision-making [30-32]. Patients are increasingly turning to online resources to guide their health care choices, and the lack of clear and consistent KTR selection criteria on transplant center websites can lead to confusion about their transplant candidacy or center-specific suitability [30,33]. This lack of access and transparency may undermine patients’ and their families’ autonomy in taking timely and informed decisions regarding their transplant care [7].

Although the lack of transparency in the KTR selection criteria is widely acknowledged [7], this study identified and quantified the gaps in the available information that can guide improvements. Policies such as the Presidential Executive Order on Advancing American Kidney Health and Centers for Medicare and Medicaid Services (CMS) initiatives increased transplant referrals and tied dialysis facility transplant rates to quality metrics [12,34]. However, to maximize the benefits of these efforts, policy adaptations, such as the IOTA model [12], which emphasized strategies that enhance transparency in the referral process, are important for improving equity and efficiency [19,35]. However, the transparency provisions initially proposed in the IOTA were ultimately eliminated. Patients often lack sufficient information about the transplantation process, which may lead them to seek evaluation at centers that are unlikely to list them, limiting their overall access to transplantation [36]. Transparency in program-specific KTR selection criteria would also benefit nephrologists, who play a crucial role in referring patients to transplant centers, and would enhance shared decision-making [4,19,37]. If nephrologists had access to the specific KTR selection criteria for each transplant center, they would have been better equipped to refer patients to the center most suited to their individual needs. Equally important is clarifying what type of information is most useful to patients. Insufficient information can hinder patient decision-making, and the information provided needs to be accurate, clear, and useful for patients. The transplant centers should clearly disclose their KTR selection criteria, ensuring that patients have the option to choose access to information, while still allowing them to disregard this information if they prefer. Such a format and content will also need refining based on stakeholder input.

Our findings aligned with prior research, which highlighted the challenges patients face in locating relevant information to choose a transplant center [17]. Moreover, significant variation in prewaitlisting practices and access to KTR selection criteria information across geographic regions and centers further complicates the decision-making process, as patients may encounter inconsistent or incomplete data depending on where they seek care [17,38,39]. However, this study was the first to provide a US national-level analysis quantifying the gaps in the availability of transplant center information on their websites. By shedding light on these barriers to accessing KTR selection criteria information, this study supported the recommendations of the Organ Procurement and Transplantation Network Ethics Committee for improving transparency in transplant program selection [7]. Access to and transparency in KTR selection criteria can be assessed using frameworks such as Accountability for Reasonableness, which emphasizes fairness and equity in health care decision-making [40,41]. Furthermore, decisions about patient candidacy tend to be more acceptable to all parties when the process is perceived as transparent [42]. Further research is needed to examine how differences in online KTR selection criteria reporting between transplant centers can impact evaluation completion and listing practices.

Beyond the gap in online patient-level information, disparities also extend to the kidney transplant referral process itself [43]. Many patients referred for transplant evaluation do not complete the necessary steps, which may stem from unclear health care provider communication and misinformation [1]. Although platforms such as the Scientific Registry of Transplant Recipients provide important data on transplant center outcomes [44], they do not usually include details on the KTR selection criteria. This highlights the ongoing need for a more transparent and regulated pretransplant evaluation process, which is crucial for building trust throughout the transplant process [1,7,19,45].

Although transplant centers generally follow protocol-driven selection processes to assess the suitability of potential candidates [46], there is heterogeneity in how KTR selection criteria are shared with patients across centers. Although some disclose financial criteria (the most frequently mentioned), others do not. Medical factors, namely clinical cutoffs, are mentioned in less than one-fifth of the center websites. Few centers report lifestyle and psychosocial factors that are decisive in transplant candidacy [11,47]. Government agencies, such as the Health Resources and Services Administration and the National Academies of Sciences, Engineering, and Medicine, are advocating for strategies to clarify the kidney transplant prewaitlist process [8,48]. We suggest that mandatory and regulated disclosure of consistent KTR selection criteria across centers’ online patient-level information could be a potential pathway to ensure more equitable access to kidney transplantation.

This study provides relevant insights into the transparency of KTR selection criteria information in a novel way using AI and NLP to facilitate broader-scale understanding. Previous research in nephrology has demonstrated that NLP is effective in identifying the presence or absence of qualitative data within large datasets, such as electronic health records [49]. However, with any AI-driven analysis, balancing sensitivity and specificity is challenging. Our model performed well overall, but it may have missed or inaccurately identified information in certain instances, as is common with AI-NLP approaches. Therefore, these results should be interpreted as an aggregate estimate of the available information rather than a definitive representation of every transplant center’s criterion.

Many factors influence transplant eligibility, and standardizing thresholds can be challenging, particularly when certain patient conditions may make eligibility more nuanced, requiring a case-by-case assessment [50]. Selection criteria may vary across centers, which may reflect differences in their experiences, such as older age, life-threatening conditions, and higher BMI. These variations in thresholds can be valuable, as some centers are more permissive than others and may include patients who might otherwise be excluded. Despite this, transparency remains the main pillar to ensure that patients can identify appropriate programs efficiently.

Moreover, despite this complexity, the reporting of KTR selection criteria, including absolute contraindication, is valuable. These criteria could be presented with explanations, such as highlighting that certain conditions may be acceptable if optimized or corrected. Disclaimers emphasizing that the information serves as a guide to help patients navigate the system and choose the transplant center most suited to their needs could also be included. We believe that having online patient-level available criteria is crucial in helping patients and providers navigate the prewaitlist process and a priority in improving the transplant system overall by increasing their chance to complete a transplant evaluation successfully.

Finally, it is relevant to note that not all centers may keep their websites up to date, and the KTR selection criteria may be available to patients through other modalities, such as paper or patient portals. Nonetheless, these alternatives are not equally accessible to all patients and may further contribute to perceived inequities in the system. In future research, it would be valuable to assess how centers make this information available through paper or patient portals and how consistent it is. Transplant center websites may be primarily created for marketing purposes with varying input from medical physicians; therefore, collaboration between medical and administrative teams is crucial to improving the accuracy and utility of patient-level online resources, ensuring that the information provided is accessible and reliable. We also acknowledge that variation in information disclosure may be influenced by center-specific factors, such as size, staffing capacity, or institutional strategy. Although our analysis focuses on the patient perspective—assessing whether information is readily accessible—future research could examine how these organizational characteristics shape transparency practices across regions.

As illustrated by the representative quotes from online patient-level information, there is variability in how centers present their KTR selection criteria. Therefore, evaluating the quality and accuracy of the websites’ content is warranted to examine specific differences across centers in online patient-level available information regarding KTR selection criteria. To better understand the heterogeneity between centers’ reporting, the use of NLP could be refined to identify and analyze more nuanced aspects of the KTR selection criteria.

### Conclusions

In summary, we found that 97.4% of online patient-level information regarding the KTR selection criteria was unavailable across centers in the United States. The extent of this lack of transparency profoundly limits patients in choosing their most suitable transplant center. Despite current federal initiatives, it remains a challenge to report specific selection criteria for KTR that could increase patients’ access to information related to their candidacy. This fosters their autonomy and enables them to make informed decisions when choosing their transplant program, ultimately impacting their access to transplants.

## Data Availability

The datasets generated or analyzed during this study are not publicly available due to the scale of the data collection, which involved scraping over 10,000 web pages and processing more than 9 million words, making it not feasible to release the full dataset. However, all data were obtained from publicly available online information given by the kidney transplant centers’ websites, and our dataset is available from the corresponding author on reasonable request.

## Appendix

Multimedia Appendix 1 Overview of study workflow and results.

## Abbreviations

AI: artificial intelligence
IOTA: Increasing Organ Transplant Access
KTR: kidney transplant recipient
LLM: large language model
NLP: natural language processing
