# Supplementary material for: Examining Transparency in Kidney Transplant Recipient Selection Criteria: Nationwide Cross-Sectional Study
Source: JMIR AI. 2025 Nov 4;4:e74066. doi: 10.2196/74066 (PMC12627972; doi:10.2196/74066)
Supplement: Multimedia Appendix 1 [file ai_v4i1e74066_app1.doc]

Supplementary Material

**Supplemental Table 1.** Key Factors for Decision-Making in Kidney Transplant Eligibility and Evaluation

**Supplemental Table 2.** Reasons for Failure in Web Scraping or LLM Data Processing of Transplant Center Websites

**Supplemental Table 3.** Representative Quotes from Websites on Kidney Transplant Recipient Selection Criteria

**Supplemental** Table 1. Key Factors for Decision-Making in Kidney Transplant Eligibility and Evaluation

| Family and/or social support system | Patients without current social support can be considered if they can care for themselves and have a support plan in place before transplantation. |
| --- | --- |
| Adherence | Transplant evaluation should be delayed if ongoing nonadherent behavior persists despite counseling. |
| Psychosocial assessment | Psychosocial assessment is recommended for all candidates to assess transplant suitability. A multidisciplinary team, including a transplant physician, surgeon, and psychosocial expert, should evaluate the candidate. |
| Support networks | Candidates without current social support can be considered for transplantation if they can care for themselves and have a support plan in place prior to transplantation. |
| Kidney function | All patients with CKD G4-G5 (GFR < 30 ml/min/1.73 m²) expected to progress to ESKD should be considered for transplantation. Specific conditions (e.g., FSGS, MN, IgAN) should not exclude candidates, but recurrence risks must be discussed. Pre-emptive kidney transplantation (living or deceased donor) is recommended for patients with eGFR <10 mL/min/1.73m² in adults, <15 mL/min/1.73m² in children, or earlier with symptoms. One center mentioned creatinine clearance* as a selection criterion in our initial manual search; therefore, it was assessed as an individual selection criterion in this study. |
| HIV | Patients with controlled HIV infection should not be excluded but should be managed in centers with experience in HIV. |
| Infection | Kidney transplantation should be delayed until active infections (bacterial, fungal, viral [except hepatitis C], parasitic) are treated. Tuberculosis should be treated prior to transplantation. Dental/periodontal disease should be evaluated before transplant. |
| Frailty | Frailty should be taken into account when assessing eligibility for kidney transplantation. Frailty should be evaluated both at the time of transplant evaluation and while on the waitlist to help guide risk management and support pre-operative rehabilitation |
| Drug/Alcohol abuse | Transplant evaluation should be postponed until substance use disorder is adequately managed. Candidates with psychiatric conditions or substance use disorders should be referred for pre-transplant counseling and support services. |
| Malignancy | Active malignancy should be treated before transplant, with exceptions for indolent cancers (e.g., prostate cancer with Gleason score ≤ 6, incidental renal tumors ≤ 1 cm). |
| Smoking | Candidates should abstain from tobacco use at least 1 month prior to wait-listing or living donor transplantation. All candidates should avoid tobacco products indefinitely after transplantation. |
| Psychiatric Illness | Delay evaluation for candidates with unstable psychiatric disorders that impact decision-making or post-transplant risk. |
| Diabetes | Candidates with ESKD and type 1 diabetes should be considered for simultaneous pancreas-kidney transplantation where available. |
| Heart disease | Do not refer patients with severe, uncorrectable, symptomatic cardiac disease or active symptomatic cardiac disease (e.g., angina, arrhythmia, heart failure) for kidney transplantation without cardiology evaluation. For those with severe cardiac conditions (NYHA Class III/IV, ejection fraction <30%), consider combined heart-kidney transplantation. Candidates with myocardial infarction should be assessed by a cardiologist before proceeding with the transplant. |
| Peripheral vascular disease | Delay transplant evaluation in patients with active symptomatic peripheral arterial disease or non-healing extremity wounds with infection. Do not exclude patients with prior aorto-iliac procedures (e.g., stent placement) if there is sufficient native artery for anastomosis. Exclude patients with severe aorto-iliac or distal vascular disease unless survival estimates meet national standards. |
| Bone and mineral disorders | Delay transplant evaluation in patients with severe hyperparathyroidism until treated. |
| Liver disease/GI disease | Do not refer patients with decompensated cirrhosis for kidney alone transplant (consider combined liver-kidney transplant). Delay kidney transplant in candidates with active symptomatic conditions (e.g., peptic ulcer, diverticulitis, pancreatitis, gallstone disease, inflammatory bowel disease) until symptoms resolve. Do not exclude candidates with a history of acute or chronic pancreatitis, asymptomatic cholelithiasis, or a history of inflammatory bowel disease. |
| Lung disease | Exclude candidates with severe irreversible obstructive or restrictive lung disease from kidney transplantation. |
| Neurological and Cognitive Disorders | Exclude candidates with progressive central neurodegenerative diseases or recent stroke/TIA. Wait 6 months post-stroke, 3 months post-TIA before transplantation. Perform neurocognitive assessments in pediatric candidates who had ESKD before age 5 or school-age children with academic difficulties. |
| Hematologic disorders | Exclude kidney transplant candidates with active multiple myeloma, AL amyloidosis (significant extrarenal involvement), acute leukemia, or high-grade lymphoma. For remission, consider with hematologist consultation. For myelodysplastic syndromes, chronic leukemia, and low-grade lymphoma, consult hematologist for transplant candidacy. |
| Immunologic assessment | Inform candidates about access to transplantation based on blood type and histocompatibility results. Offer immunologically reduced access to larger donor pools, kidney exchange, or desensitization. |
| Age | Consider age along with comorbidities like frailty in transplant eligibility. Do not exclude due to age alone. |
| BMI (kg/m2) n (%) | Obesity should not be the sole reason for excluding candidates from kidney transplantation. Weight loss interventions should be offered to candidates with obesity prior to transplantation. |
| Dialysis | Refer dialysis patients for transplant evaluation once stable and kidney failure is irreversible. For candidates not yet on dialysis, refer 6 to 12 months before anticipated initiation to allow for living donor identification and potential pre-emptive transplantation. |
| Mobility | While not an explicit criterion, mobility impacts transplant eligibility. Mobility is an important factor in evaluating a patient's overall health and ability to manage post-transplant care, and severe mobility issues may lead to exclusion based on concerns about surgical recovery and long-term outcomes. |
| **Other factors**^a^ | |
| Finances/Health insurance | Financial status/health insurance are important factors for transplant eligibility. While KDIGO guidelines do not explicitly mention these criteria, transplant centers often assess financial aspects as part of a broader social evaluation. |
| Homeless or other unstable living conditions | Homelessness or unstable living conditions are not formal exclusions, but lack of stable housing or support systems may hinder a patient’s ability to manage transplant demands and affect long-term success, potentially leading to delays or exclusion from transplant consideration. |
| Life-threatening diseases | While not explicitly listed as exclusion criteria, KDIGO emphasizes evaluating comorbid conditions. Severe non-renal diseases or malignancies that could worsen with immunosuppression are often excluded due to high risks and poor outcomes. |
| Multiple organ failure | Patients should undergo a comprehensive evaluation of their overall health and organ function, as multiple organ failure typically indicates a poor prognosis and may result in exclusion from transplantation. |

Adapted from KDIGO Clinical Practice Guideline on the Evaluation and Management of Candidates for Kidney Transplantation, 2020. *Note*: ^a^Not explicitly mentioned by KDIGO Guidelines. *Abbreviations*: CKD, Chronic Kidney Disease; G4-G5, Stage 4 and Stage 5; ESKD, End-Stage Kidney Disease; HIV, Human Immunodeficiency Virus; FSGS, Focal Segmental Glomerulosclerosis; MN, Membranous Nephropathy; IgAN, IgA Nephropathy; NYHA, New York Heart Assocation; GI, gastrointestinal; TIA, Transient Ischemic Attack; AL amyloidosis, Amyloid Light Chain Amyloidosis; eGFR, estimated Glomerular Filtration Rate; BMI, Body Mass Index.

**Supplemental Table 2. Reasons for Failure in Web Scraping or LLM Data Processing of Transplant Center Websites**

| **Reason** | **Number of Centers** |
| --- | --- |
| Duplicate entries by host^a^  Access denied: Security restrictions  No keyword match: Kidney/transplant  No kidney transplant webpage found  Payment required  Authorization required | 33  9  7  1  1  1 |

^a^“Duplicate entries by host” means that multiple transplant centers were associated with the same website (e.g., va.gov). Since grouping was performed at the host level, only one center was included, and the rest were excluded from web scraping. This often occurred with aggregator sites that list multiple centers under a single domain.

**Supplemental** Table 3. Representative Quotes from Websites on Kidney Transplant Recipient Selection Criteria

| **Criteria** | **Representative Quotes** |
| --- | --- |
| Age | “You may not be a good candidate if you are above a certain age ...”  “One-third of our transplant patients are over the age of 65.” |
| GFR | “Potential kidney transplant patients should be referred when their estimated glomerular filtration rate (eGFR) is nearing 20-30 ml/min.”  “You can qualify to be on the transplant list if your kidneys are functioning at 20 mg/dl or less.” |
| CKD stage | “We start the living donor kidney transplant process in preemptive care when a patient is in early stage kidney disease.”  “You may need a kidney transplant if you have end-stage renal disease (ESRD). This is a permanent condition of kidney failure. It often needs dialysis.” |
| Dialysis | “Some people who need dialysis on a permanent basis might be candidates for a kidney transplant.”  “Those with end-stage renal disease may be eligible for a kidney transplant, whether or not they are on dialysis. In fact, the sooner a kidney failure patient undergoes transplant, the better.” |
| Finances/  Health Insurance | “You need the financial resources to cover your transplant and the immunosuppression after.”  “All transplant patients are advised to make long-term financial plans for transplant. Patients whose financial or insurance situation is uncertain are frequently recommended to engage in fundraising efforts to establish a "safety net" of funds they can access for post-transplant costs.” |
| Life-threatening diseases | “You might be considered a good candidate if you don't have significant heart, lung, or liver disease or other diseases, such as cancer, which might decrease your life span.”  “You need to meet certain guidelines to be eligible for transplant. Those who have the following are not typically eligible: Certain infections, such as TB, bone infections or hepatitis Inability to take multiple daily medications Recent history of cancer Heart, lung or liver disease Mental illness Life-threatening diseases High-risk behaviors such as smoking, alcohol and drug abuse, etc.” |

*Abbreviations*: BMI, Body Mass Index; GFR, Glomerular Filtration Rate; U.S., United States; CKD, chronic kidney disease.
